# Fertility Preservation in Cancer Patients During the Coronavirus (COVID-19) Pandemic

**DOI:** 10.3389/fonc.2020.01009

**Published:** 2020-06-04

**Authors:** Miriam Dellino, Carla Minoia, Angelo Virgilio Paradiso, Raffaella De Palo, Erica Silvestris

**Affiliations:** ^1^Gynecologic Oncology Unit, IRCCS Istituto Tumori “Giovanni Paolo II”, Bari, Italy; ^2^Haematology Unit, IRCCS Istituto Tumori “Giovanni Paolo II”, Bari, Italy; ^3^Institutional BioBank, Experimental Oncology and Biobank Management Unit, IRCCS Istituto Tumori “Giovanni Paolo II”, Bari, Italy

**Keywords:** COVID-19, cancer patients, fertility preservation, gonadotoxicity, pandemic

## Abstract

The severe acute respiratory syndrome coronavirus 2 (SARS-CoV-2), also identified as Corona virus disease 19 (COVID-19), has recently produced a dramatic and widespread sanitary emergency. However, despite the necessity to assist a substantial number of affected patients, it is also essential to, at the same time, guarantee the usual clinical care, particularly to cancer patients, including fertility preservation (FP) strategies before the beginning of the anti-cancer treatments. The FP techniques for adult female patients include oocyte and embryo cryopreservation, which require both adequate ovarian reserve (OR) and controlled ovarian stimulation (COS) to promote multiple follicular growth. However, ovarian tissue cryopreservation is an additional FP practice suitable when an anti-cancer treatment is urgently required, whereas, for male patients, sperm cryopreservation is a simple and well-adopted procedure. Here, we focus on the current conditions in terms of agreements and rules of FP procedures during this COVID-19 pandemic to achieve and provide useful recommendations for the adoption of these techniques in patients with cancer.

## Introduction

The diffusion of the Severe Acute Respiratory Syndrome Coronavirus 2 (SARS-CoV-2), or Coronavirus Disease 19 (COVID-19), has rapidly become a health emergency of global proportions. The virus, primarily discovered in China to be responsible of a severe interstitial pneumonia (1, 2), has subsequently revealed its contagiousness in countries outside of China, such as Italy, thus leading the World Health Organization (WHO) to define this infection as a real pandemic (3).

It has been reported by the Italian National Institute of Health (Istituto Superiore di Sanità, ISS) that Italian COVID-19^+^ patients have a median age slightly older than 60, and male prevalence is as high as approximately 48.6%, although 25% of subjects under 45 yrs, namely in the reproductive age, are also affected, with a prevalence of a little <15% for females subjects (4). The May 14 report from mass media concerning COVID-19 surveillance describes 31,368 deaths, including ~1.5% of people younger than 50 yrs.

Several concurrent pathologies, such as diabetes, obesity, and renal and cardiovascular diseases, have apparently been related to a worse outcome for COVID-19 infection (5), whereas, at present, it is unclear whether or not a concomitant cancer provides an independent risk of unfavorable or dramatic evolution of the infectious disease (6). However, preliminary data from the ISS regarding the prognosis of COVID-19^+^ cancer patients describe a mortality higher than 20% in patients with onco-hematological disorders, which is primarily ascribed to treatment-induced concurrent immunosuppression as a major cause influencing the cancer prognosis (7). Indeed, in a recent multivariate analysis exploring the evolution of virus infection in patients with other cancers (5), the oncologic condition is not regarded as an independent risk factor of worsening prognosis of COVID-19 disease, although until today only a few reports have focused this topic.

Despite such preliminary—though incomplete—information, the Italian Association of Medical Oncology (AIOM), in partnership with the boards of both Academic Oncologists (COMU) and the Oncology Units' Directors (CIPOMO), has recently advised specific management recommendations for oncologic patients (8); they suggest delaying anti-cancer treatments in non-urgent clinical settings while at the same time respecting conditions of clinical urgency, such as neo-adjuvant treatments that cannot be delayed (9). To this regard, the fertility preservation (FP) procedures available for cancer patients desiring to restore their reproductive potential after the cancer healing must be considered as a topic of relevant interest during the COVID-19 crisis. In fact, although, under normal conditions, the embryo/oocyte cryopreservation as well as the ovarian cortex cryostorage are established female FP approaches, and sperm freezing and cryopreservation is a validated procedure in males, during the COVID-19 pandemic, it is necessary to define the potential utilization and safety of these procedures for cancer patients in their oncologic programs. Thus, in line with the anti-COVID-19 assessment from the ISS, the National Transplant Centre (NTC) has endorsed some recommendations implying the prosecution of the FP programs, particularly for the cryopreservation procedures, with a careful concomitant evaluation of the existing COVID-19 symptoms. Therefore, a preventive triage of patients with fever and/or minimal or mild respiratory symptoms is essential to avoiding exposure to other patients including those addressed to FP programs as well as to dedicated healthcare providers (10).

## Fertility Preservation Techniques in Cancer Patients

The innovative integrated anti-cancer treatments are undoubtedly efficient and produce improved responses with increased survival rates, thus allowing a large number of patients a chance to have a family following the cancer healing. Therefore, for these patients, the Public Health Service should warranty the availability of appropriate and urgent FP procedures in concomitance with the anti-cancer programmed treatments, even during the current COVID-19 health emergency.

The International guidelines (11–14) for fertility preservation in cancer patients established that reproductive counseling should be closely proposed after diagnosis and staging of the disease to detect better strategies for reproductive safety according to the different tumor prognoses.

Both the American Society of Clinical Oncology (ASCO) and the European Society for Medical Oncology (ESMO) recommend the cryopreservation of both mature oocytes and embryos in women with an adequate ovarian reserve (OR) and after controlled ovarian stimulation (COS) to induce the multiple follicular growth (MFG). However, to avoid the hormonal stimuli, it is also alternatively suggested to cryopreserve immature oocytes after their ovarian retrieval since the oocytes are not greatly damaged by the freezing injury and are metaphase spindle free (15). When the COS-induced MGF, which usually requires about 9–15 days, is not achievable, as in pre-pubescent patients, or not allowable for therapeutic urgent needs, the ovarian tissue cryopreservation is an alternative procedure that can be proposed (16). To perform the ovarian tissue sampling, however, it is necessary to plan a laparoscopic surgery and subsequent cryopreservation of the cortex fragments until the next orthotopic or heterotopic reimplantation, namely, on the residual ovary or in a peritoneal pocket. On the other hand, gonad shielding during radiation therapy as well as ovarian temporary suppression of gonadotropin-releasing hormone (GnRH) agonists (16) and other available procedures to preserve ovarian function are also considered functional. With respect to the controversial utilization of GnRHa before anti-cancer treatments, several Authors describe this procedure as a suitable method in BC patients requiring urgent chemotherapy protocols (17), while it appears improper in patients with lymphoproliferative diseases (18, 19).

Concerning the male patients, the semen cryopreservation is a largely adopted FP procedure that, if properly programmed, does not imply any delay in starting anti-cancer treatments. However, at present, several groups are interested in the optimization of testicular tissue reimplantation previously cryopreserved, although this procedure has not yet been tested in humans (20).

## Assisted Reproduction Technology (ART) and Fertility Preservation (FP) During the Covid-19 Pandemic

The COVID-19 pandemic has brought distinctive challenges to the global healthcare community for the dramatic worldwide escalation of morbidity and mortality.

The British Royal College of Obstetricians & Gynecologists (RCOG) (21) and the American College of Obstetrics and Gynecology (ACOG) (21) have proposed only a few suggestions for pregnant COVID-19^+^ patients based on the limited data reported in literature. In fact, despite the feared risk of infection as well as increasing morbidity and possible adverse perinatal outcomes in infected pregnant women (22, 23), very few data or scientific information are today available on cautionary and protective measures to be adopted for the ART procedures in cancer patients. In young cancer patients urgently undergoing gonadotoxic chemo- and/or radiotherapy protocols, the FP programs require an ethical priority and cannot be delayed or suspended in relation to the putative increased risk of infection (24). Therefore, in order to consent to these patients to receive FP techniques and carry on their parental project at the cancer healing, a specified counseling with both oncologists and oncofertility specialists should in single cases evaluate the realistic risk of COVID-19 infection to adopt precautionary procedures for preventing the virus disease (24).

Occurrence of COVID-19^+^ infection during the COS should, however, suggest the cancellation of the FP program, whereas when the oocyte pick-up has been already completed, the egg cryostorage is pursuable for an embryo transfer—at least until the condition is proved to be disease free. Therefore, the ART Units probably need to implement proper rules in their organization to warrant programmed treatments to COVID-19^+^ subjects in addition to conventional precautions and prevention measures, and, for the lack of defined guidelines, access to ART procedures has been restrained, particularly in geographic areas of high virus diffusion and morbidity as well as in several regions of northern and central Italy. Therefore, besides the utility of autonomous solutions to assist COVID-19^+^ people by ART centers, it is essential that both health authorities and scientific societies share and adopt defined and standardized criteria and rules.

During COVID-19 pandemic, the European Society of Human Reproduction and Embryology (ESHRE) recommends that all healthy and fertile women planning a natural pregnancy, even in the absence of COVID-19 infection, should avoid their maternity project at this time, while for infertile patients undergoing ART treatment it would be preferable to postpone the procedures based on oocyte or embryo cryopreservation. Furthermore, the American Society for Reproductive Medicine (ASRM) suggests, for healthy subjects, the interruption of any ART treatment, including the gamete cryopreservation, as well as the suspension of embryo reimplantation and elective surgery, while maintaining the cryopreservation procedures for cancer patients urgently undergoing COS (25).

In prepuberal and adult patients affected by hormone-sensitive cancers and urgently requiring gonadotoxic protocols, the ovarian cortex cryopreservation for future reimplantation appears to be a suitable procedure to prevent the delay of cancer treatment, though COVID-19 testing appears to be necessary for patients before addressing the pelvic surgery to obtain the ovarian tissue. Similarly, the ovarian transposition, a FP procedure suggested before starting pelvic radiation therapy, should require COVID-19 diagnostics before its application in females with cancer, whereas the temporary ovarian suppression of GnRH agonists during anti-cancer treatment is a protective procedure for the ovarian function and is also pursuable during this pandemic once COVID-19 testing has been assessed in patients (24).

However, based on the short time since the COVID-19 emergence, a major question concerns the few data disseminated from the relevant scientific societies on the proposal of a shared consensus on either the prosecution or interruption of ART procedures in cancer patients. There should be, indeed, no reason to consider them at a higher of infection once well-defined precautions have been adopted. On the other hand, the postulated risk of disease transmission in gametes by infected people has not been definitely assessed, and, at least in males, the presence of the virus in seminal fluid has not been confirmed (23), thus suggesting that sperm from COVID-19^+^ cancer males do not contain the virus and that their cryopreservation could be regularly adopted for FP programs.

By contrast, there are no data evaluating the risk of a possible transmission of COVID-19 to oocytes in infected women after their COS and oocyte recruitment following the MFG. Therefore, the lack of information related to this specific topic cannot support or deny the suitability and safety of ART procedures in infected cancer females.

The only study available includes a meta-analysis from Mullins et al. describing the impact on COVID-19 infected women during their pregnancy. The authors reported that from 32 affected women, seven of them gave birth to asymptomatic newborns, while two babies were assisted after birth in an intensive care unit (ICU), and two more babies died for other reasons; none of the newborns died from COVID-19^+^-related issues (26). News from mass media over the last weeks also reported the birth of at least two COVID-19-negative newborns from positive mothers in Lombardy, whereas 20 asymptomatic virus-positive newborns have been registered at the National Health Ministry. However, this suggests that, at present, there is no evidence for putative transplacental transmission of the infection.

Nonetheless, the available data are inadequate to support the wished inability of both males and females COVID-19^+^ cancer patients to transfer the infection to their own gametes, and more studies are necessary to assess these questions in planning the FP activities to prevent the viral transmittance to fetuses.

## The Covid-19 and the Cancer Patients' Prognostic Evaluation

It has been reported in literature that the initial symptoms of the COVID-19 infection, such as cough, fever, shortness of breath, muscle pain, chest pain, headache, and diarrhea, are similar to those occurring in cancer patients after treatment protocols using chemo, targeted, and immune therapy (27), and patients are more susceptible to the COVID-19 infection due to exhaustion of their immune response resulting from cancer. Therefore, the COVID-19 pandemic has also posed several challenges to the clinical practice of oncologists, requiring at this time an accurate and adequate screening for anamnestic information concerning typical aspects of the viral infection as well as for all respiratory and systemic symptoms that these patients may manifest (28).

In fact, it has been reported by Liang et al. that the risk of COVID-19 infection is definitely higher in cancer patients as an effect of their clinical condition of increased morbidity, which rapidly result in worsening and the occurrence of severe complications requiring intensive therapy care, especially as compared to other non-cancer patients (29).

Therefore, it is absolutely necessary during the FP counseling for cancer patients to triage for COVID-19 in accordance with local guidelines, which also includes a nasopharyngeal buffer for the screening of the infection for its potentially impact on the prognosis. However, at present, despite the absence of studies investigating cancer patient candidates to FP procedures of a fertile age, this standardized screening procedure, which is largely used today when facing the infection, should be performed.

## Conclusion

The FP is an essential opportunity for cancer patients requiring gonadotoxic treatment and the desire to produce a family following cancer recovery.

Therefore, despite the very few published data on the FP topic in COVID-19^+^ cancer patients, the virus transmittance to either sperms or oocytes is apparently an averted biologic event, and the cryopreservation of gametes can be maintained in these patients following the necessary precautions against the infection. In these situations, it is critical to have dedicated triage and to respect all the imposed limitations to avoid the increase of the infection. On the other hand, it is also necessary for all ART and other centers enrolled in FP programs to adopt uniformly regulated rules when managing cancer patients undergoing those procedures to avoid the risk of infection or reveal its presence in apparently asymptomatic patients. To this, it is also necessary that health authorities and scientific societies take an interest in this topic and provide regulatory standards.

In conclusion, while it is possible to reassure these patients that their gametes are not infected by COVID-19 and can thus be processed for cryopreservation, a screening by serum detection of antibodies and COVID-19 proteins, or a nasopharyngeal pad, should be mandatory in cancer patients participating in FP programs.

## Author Contributions

MD reviewed the literature and wrote the manuscript. CM contributed to literature review. AP contributed to writing. RD and ES contributed critically reviewed the manuscript. ES reviewed the literature and wrote the manuscript. All authors have read and agreed to the published version of the manuscript.

## Conflict of Interest

The authors declare that the research was conducted in the absence of any commercial or financial relationships that could be construed as a potential conflict of interest.
